# Flexible FLIG-Based Temperature Sensor Enabled by Femtosecond Laser Direct Writing for Thermal Monitoring in Health Systems

**DOI:** 10.3390/s25154643

**Published:** 2025-07-26

**Authors:** Huansheng Wu, Cong Wang, Linpeng Liu, Ji’an Duan

**Affiliations:** State Key Laboratory of Precision Manufacturing for Extreme Service Performance, College of Mechanical and Electrical Engineering, Central South University, Changsha 410083, China

**Keywords:** laser direct writing, temperature sensor, laser-induced graphene, flexible electronics

## Abstract

In this study, a facile and mask-free femtosecond laser direct writing (FLDW) approach is proposed to fabricate porous graphene (FLIG) patterns directly on polyimide (PI) substrates. By systematically adjusting the laser scanning spacing (10–25 μm), denser and more continuous microstructures are obtained, resulting in significantly enhanced thermal sensitivity. The optimized sensor demonstrated a temperature coefficient of 0.698% °C^−1^ within the range of 40–120 °C, with response and recovery times of 10.3 s and 20.9 s, respectively. Furthermore, it exhibits remarkable signal stability across multiple thermal cycles, a testament to its reliability in extreme conditions. Moreover, the sensor was successfully integrated into a 3D-printed robotic platform, achieving both contact and non-contact temperature detection. These results underscore the sensor’s practical adaptability for real-time thermal sensing. This work presents a viable and scalable methodology for fabricating high-performance FLIG-based flexible temperature sensors, with extensive application prospects in wearable electronics, electronic skin, and intelligent human–machine interfaces.

## 1. Introduction

In recent years, flexible electronic devices have garnered increasing attention owing to their excellent mechanical flexibility, lightweight construction, and outstanding sensing capabilities [1,2,3,4,5,6,7,8]. These devices have found broad applications in diverse fields, including wearable health monitoring [9,10], robotic tactile interfaces [11,12], and real-time environmental sensing [13]. A wide range of flexible sensors has been developed to detect various physical stimuli such as pressure [14], strain [15], vibration [16], and temperature [17,18]. Within such systems, the selection of an appropriate substrate material is critical to ensure structural integrity, long-term stability, and process compatibility. Polyimide (PI) is a representative flexible polymer that offers high mechanical strength, excellent bendability, and thermal resistance, withstanding temperatures above 300 °C, as well as chemical stability, electrical insulation, and low-cost availability [19]. As a result, PI films have been extensively employed in flexible electronics, particularly in sensor devices operating under elevated-temperature conditions [20,21,22].

Among the various functional materials for flexible sensors, graphene has emerged as a highly promising candidate for the development of flexible temperature sensors due to its outstanding electrical conductivity, high carrier mobility, and excellent thermal properties [23,24,25]. However, the scalable, cost-effective, and environmentally benign fabrication of graphene-based flexible sensors remains a considerable challenge. Conventional techniques, including chemical vapor deposition (CVD) and chemical reduction in graphene oxide, generally necessitate elevated temperatures, hazardous chemical reagents, or intricate transfer procedures [26,27,28]. These processes are not only incompatible with flexible polymer substrates but also challenging to implement on a large scale in industrial manufacturing settings.

As an emerging digital fabrication technology, femtosecond laser direct writing (FLDW) has attracted increasing attention due to its capacity to rapidly and mask-free fabricate functional materials [29,30,31]. Specifically, femtosecond laser-induced graphene (FLIG) can be engineered with precision by regulating key laser parameters, including pulse energy, wavelength, scanning speed, and scanning spacing [32,33,34]. Compared to continuous-wave and long-pulse laser sources, femtosecond lasers offer distinct advantages for the carbonization and graphitization of polymer substrates. The ultra-short pulse width and extremely high peak power facilitate nonlinear absorption and localized photothermal effects, which induce rapid pyrolysis and high-quality graphene formation with minimal thermal damage. These attributes are particularly beneficial for flexible substrates such as polyimide. Recent studies have demonstrated that femtosecond laser direct writing enables the formation of porous graphene structures with excellent conductivity, mechanical robustness, and integration compatibility, outperforming traditional infrared laser systems in resolution and processing control [35,36]. This localized photothermal process enables the direct carbonization and reduction in polymer surfaces, leading to the formation of conductive graphene microstructures [37]. In comparison with conventional graphene synthesis methods, the FLIG process offers several distinct advantages, including the ability to operate under ambient conditions, the capacity for flexible patterning, high spatial resolution, and broad substrate compatibility. Additionally, FLIG features a high specific surface area, excellent electrical conductivity, and strong thermal responsiveness, rendering it a promising platform for constructing high-performance sensing layers [38,39].

However, there is a paucity of systematic research on the correlation between laser processing parameters (especially scanning spacing) and FLIG sensing performance. It is imperative to possess an in-depth comprehension of this mechanism to optimize the design of the device structure and the preparation strategy. In this study, femtosecond laser direct writing technology was utilized to efficiently and cost-effectively fabricate FLIG films on PI films. The effects of laser scanning spacing on the structural, electrical, and thermal properties of the films were systematically investigated. Concurrently, the fabricated flexible temperature sensors are integrated into the robotic system to enable intelligent thermal sensing, thereby further expanding their application potential in the frontier fields of intelligent robotics and human–machine interaction.

## 2. Experimental Section

### 2.1. Materials

PDMS (Sylgard 184) was obtained from Dow Corning. Anhydrous ethanol (99.7% purity) was purchased from Taicang Xintai Alcohol Co., Ltd. (Taicang, Jiangsu, China). Conductive silver paste was sourced from Shanghai Julong Electronic Technology Co., Ltd., Shanghai, China. Copper electrodes were provided by Dongguan Hengchuang Adhesive Products Co., Ltd., Dongguan, China. A commercial polyimide film was used as a flexible substrate for sensor fabrication.

### 2.2. Preparation of Flexible Temperature Sensor

Polyimide films were cut into 3 cm × 3 cm square sheets and cleaned using anhydrous ethanol to remove surface contaminants. The cleaned films were left to air dry completely at room temperature. Graphene patterns were subsequently fabricated on the PI surface using a femtosecond laser direct writing (FLDW) technique. A HR-Platform-0203 femtosecond laser system (Wuhan Huari-Precision Laser Co., Ltd., Wuhan, China) operating at a wavelength of 1030 nm, an average laser power of 1.3 w, a spot diameter of about 66 μm, a repetition frequency of 700 kHz, and a scanning speed of 150 mm/s was employed. The scanning spacing was varied from 10 μm to 25 μm in 5 μm increments. Laser pathing was controlled via a high-speed galvanometric scanner (Scanlab, Puchheim, Germany), and the FLIG structure was designed with a rectangular geometry consisting of a central sensing region and flanking electrode contact zones. Specifically, the sensing layer was patterned as a 10 cm × 10 cm square, while two 5 cm × 5 cm square FLIG regions were symmetrically fabricated on the left and right sides as electrode connection pads. This configuration enables efficient signal collection, stable thermal distribution, and mechanical flexibility. During femtosecond laser writing, the scanning path was set as a series of parallel lines with a scanning pitch ranging from 10 to 25 μm, depending on the process parameters. A focused laser spot of approximately 66 μm in diameter ensured sufficient overlap between adjacent scans to achieve continuous and uniform graphitization. After laser processing, conductive silver paste was applied to both ends of the FLIG pattern, followed by insertion of copper electrodes after the paste was fully cured. Finally, a layer of PDMS was spin-coated onto the device surface for encapsulation. The device was then placed in an oven at 65 °C for 8 h to complete PDMS curing and obtain the flexible temperature sensor.

### 2.3. Characterization and Testing

The surface morphology and elemental composition of the FLIG structures formed on PI films were characterized by scanning electron microscopy (SEM, TESCAN, Brno, Czech Republic). The basic sensing performance of the fabricated sensors, including temperature response sensitivity and linearity, was evaluated using a commercial heating platform (Shenzhen Bangyuan Electronics Co., Ltd., Shenzhen, China) and a digital multimeter (DAQ6510, KEITHLEY, Solon, OH, USA). The external contact and non-contact thermal sensing performance was further tested using a custom-built experimental setup, consisting of an electronic temperature probe (Hengshui Zhengxu Electronic Technology Co., Ltd., Hengshui, China), a 3D-printed robotic manipulator, and a hot air gun (DELIXI Electric Co., Ltd., Hangzhou, China).

## 3. Results and Discussion

**Figure 1:**
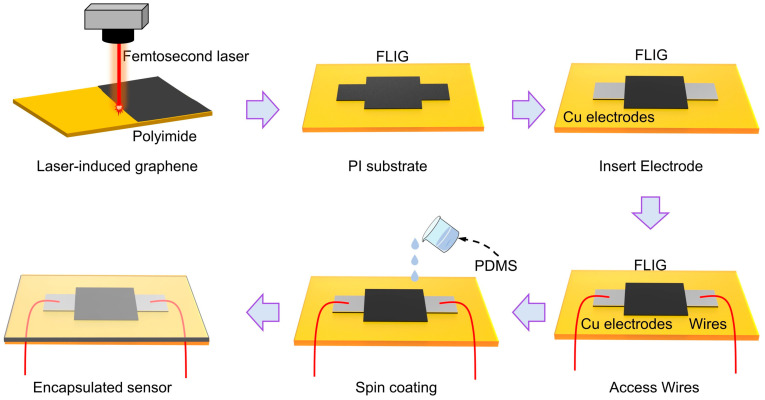
Process flow for the preparation of flexible FLIG temperature sensors based on FLDW technology.

To ensure accurate response and stable temperature sensing, the active region of the flexible sensor was designed as a rectangular pattern to facilitate uniform heat distribution. The conductive layer was formed using femtosecond laser-induced graphene (FLIG), followed by the application of conductive silver paste and the insertion of copper sheet electrodes at both ends to establish electrical connections. The core sensing unit was entirely fabricated using femtosecond laser direct writing (FLDW), a technique that offers high structural continuity, simple processing, and patterning flexibility. As illustrated in Figure 1, the preparation of a flexible temperature sensor based on femtosecond laser direct writing (FLDW) technology is a complex process. The device’s fundamental structure comprises a PI flexible substrate, a FLIG sensing layer, a silver paste electrode, a copper conductive sheet, and a PDMS encapsulation layer. The device exhibits advantageous flexibility, thermal stability, and conductivity characteristics. In the pre-processing stage, the PI film is first cut into square sheets measuring 3 cm × 3 cm. Then, the surface is thoroughly cleaned with anhydrous ethanol to remove impurities, including electrostatically adsorbed dust, oil, and particles. This ensures the cleanliness and consistency of the laser processing area. Subsequent to the cleaning process, it is imperative to allow the surface to undergo natural drying. This is to ensure that the residual solvent does not exert an adverse effect on the laser induction efficiency.

In the patterning process, computer-aided design (CAD) software was used to define the boundaries and dimensions of the FLIG patterns. These patterns were arranged in a rectangular grid to ensure uniform distribution and repeatable thermal response. A femtosecond laser direct writing system (wavelength: 1030 nm; repetition rate: 700 kHz) was then employed to pattern the surface of the PI substrate. The laser scanning speed was fixed at 150 mm/s, while the scanning pitch was varied from 10 μm to 25 μm in 5 μm increments to evaluate its influence on microstructure formation and device performance. Laser beam modulation and path control were realized using a high-speed galvanometric scanner, enabling precise energy delivery and consistent graphitization within the patterned regions. After laser processing, conductive silver paste was applied to both ends of the FLIG structure, followed by insertion of pre-cut copper sheets as electrodes. This configuration helps minimize contact resistance and improves mechanical stability. Once the silver paste was fully cured, electrical wires were soldered to complete the signal transmission path. Finally, the entire device surface was encapsulated by spin-coating a layer of PDMS, which offers dual advantages: enhanced mechanical durability and flexible adhesion, as well as good airtightness and electrical insulation. The PDMS layer was cured at 65 °C for 8 h. The overall fabrication process is mask-free and photolithography-free, and is conducted under ambient conditions, demonstrating excellent environmental friendliness and manufacturing flexibility.

**Figure 2:**
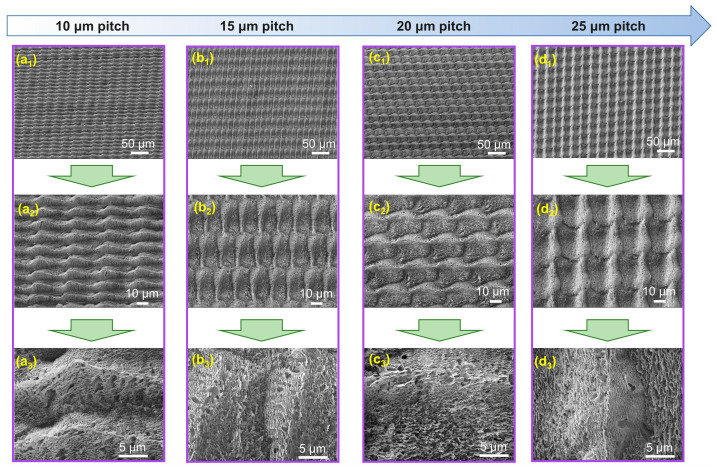
Surface morphology of femtosecond laser-induced graphene (FLIG) structures at different scanning spacings. (**a_1_**–**d_1_**) Low magnification SEM images under the corresponding conditions, demonstrating the macroscopic pattern homogeneity. (**a_2_**–**d_2_**) Medium-magnification images reveal the folded and aligned structure of the FLIG surface. (**a_3_**–**d_3_**) High-magnification images, further revealing the evolution of the microporous structure of FLIG.

On the basis of the above standardized preparation process, this study further investigated the effect of laser scanning spacing on the formation quality and microscopic morphology of FLIG structures. To ascertain the effects of varying scanning spacings (10 μm, 15 μm, 20 μm, and 25 μm) on the formation of FLIG, the surface structures of these samples were systematically compared. This was achieved by maintaining the laser power, scanning speed, and other parameters at constant levels. The evolution patterns of the samples were observed by multi-scale scanning electron microscopy (SEM), as shown in Figure 2. As evidenced by the low magnification plots (Figure 2(a_1_–d_1_)), it can be observed that a decrease in scanning pitch results in an increase in the overlapping area between the laser scanning paths, thereby forming a more continuous and denser FLIG pattern. The patterns formed under the 10 μm and 15 μm spacing conditions are uniformly aligned, and there are no obvious gaps between the carbonized regions. This indicates that the superposition of the energy of the laser acting on adjacent paths promotes the full development of the graphitization process. Conversely, when the scanning spacing was increased to 20 μm and 25 μm, evident discontinuities manifested between the FLIG patterns, characterized by distinct boundaries and a less organized arrangement between the carbonized bands. This finding suggests that sufficient structural remodeling did not occur on the PI surface within the laser-uncovered region, resulting in sparse conductive paths and diminished connectivity.

Further observation of the medium- and high-magnification images (Figure 2(a_2_–d_2_,a_3_–d_3_)) reveals the formation of a typical porous structure on the surface of FLIG. This porous morphology is the result of the rapid pyrolysis of the polymer, gas release, and carbonization contraction induced by the instantaneous heating of the femtosecond laser. Conversely, the surface of unprocessed PI is smooth with no obvious microstructure. The porous microstructure of FLIG not only significantly increases the specific surface area but also effectively regulates the thermal diffusion rate and thermal response path, thus enhancing its temperature sensitivity. Furthermore, the porous FLIG surfaces generated under 10 μm and 15 μm conditions exhibit enhanced uniformity in structure, characterized by a more compact pore size distribution and a denser stacking between graphite layers. This results in the formation of a more continuous, conductive, and thermally responsive network. The observed structural characteristics provide a significant foundation for the subsequent sensing performance. Conversely, the FLIG region generated at a 25 μm pitch exhibits inadequate carbonization, indicative of the structural distortion arising from uneven laser coverage. This structural defect will lead to the interruption of the conductive network and discontinuity of the thermal conduction path, which will ultimately result in a weakening of the response sensitivity and stability of the temperature sensor.

In summary, it is evident that the laser scanning pitch, as a pivotal process parameter for regulating the quality of FLIG structures, exerts a significant influence on the integrity and continuity of pattern forming. Furthermore, it determines the distribution state of FLIG microporous structures. A smaller scanning pitch has been shown to facilitate the formation of a dense, uniform, and well-connected porous FLIG layer, which has been demonstrated to significantly enhance the response sensitivity, linearity, and stability of the flexible temperature sensor.

**Figure 3:**
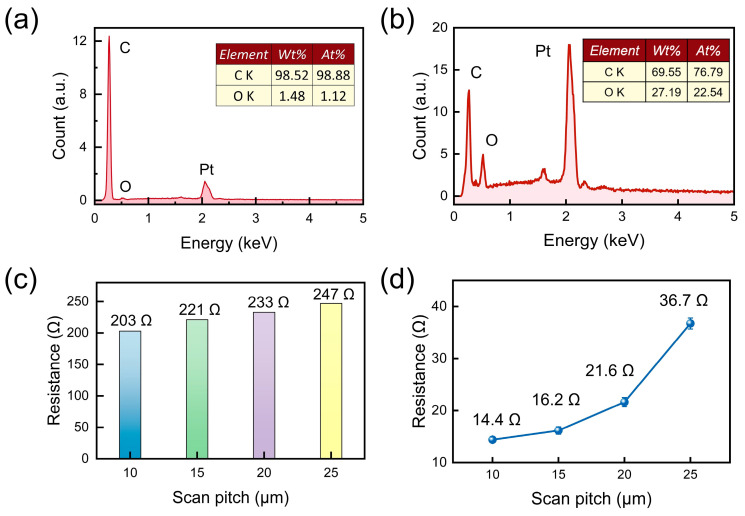
Conductivity of FLIG at different scanning spacings. (**a**) EDS elemental analysis of the PI surface after femtosecond-excitation processing at a scanning spacing of 10 μm. (**b**) EDS elemental analysis of the PI surface. (**c**) Initial resistance of FLIG at scanning spacings of 10 μm, 15 μm, 20 μm, and 25 μm, respectively. (**d**) Four-probe resistance of FLIG at scanning spacings of 10 μm, 15 μm, 20 μm, and 25 μm, respectively.

In order to further reveal the elemental composition of the FLIG structure and its electrical properties, the samples were characterized by energy spectrum analysis (EDS). As demonstrated in Figure 3a, the energy spectroscopy test was conducted at a scanning spacing of 10 μm. The results indicated that the mass fraction of carbon on the FLIG surface was as high as 98.52%, while the mass fraction of oxygen was as high as 1.48%. As illustrated in Figure 3b, the energy spectrum of the unprocessed PI substrate surface reveals that the mass fraction of carbon is 69.55%, while the mass fraction of oxygen is 27.19%. Prior to and following the femtosecond laser processing, there was an increase in the mass fraction of carbon on the PI surface of 28.97%, accompanied by a decrease in the oxygen content of 25.71%. This comparison underscores the substantial impact of femtosecond laser treatment in promoting elevated carbon content and the formation of a graphene-like structure. The results of this study demonstrate that femtosecond laser treatment effectively transforms PI substrates into conductive graphene networks.

Furthermore, Figure 3c presents the initial resistance measurements of the prepared FLIG films at varying scanning spacings (10, 15, 20, and 25 μm). The resistance value undergoes an incremental rise from 203 Ω (10 μm) to 247 Ω (25 μm) as the scanning pitch is increased. This increase in resistance value is primarily attributable to the fact that the FLIG structure becomes sparser at larger scanning spacings, thereby disrupting the continuity of the conductive network and consequently increasing the overall resistance. As illustrated in Figure 3d, the outcomes of the four-probe resistance measurements at varying scanning spacings are presented. The results demonstrate that there is an increase in resistance from 14.4 Ω to 36.7 Ω. This enhancement in resistance can be attributed primarily to the augmented scanning spacing, which consequently diminishes the laser scanning overlap area. This, in turn, results in an incomplete induction of graphene on the PI surface. Consequently, the conductive network in the FLIG region becomes inhomogeneous, resulting in elevated resistance. This result further emphasizes the critical role of scanning spacing in influencing the degree of graphitization and overall conductivity of laser-induced graphene. This finding is highly consistent with the results of the aforementioned SEM morphology analysis, which further confirms the important role of scanning pitch as a key process parameter in regulating the conductivity and microstructural integrity of FLIG.

**Figure 4:**
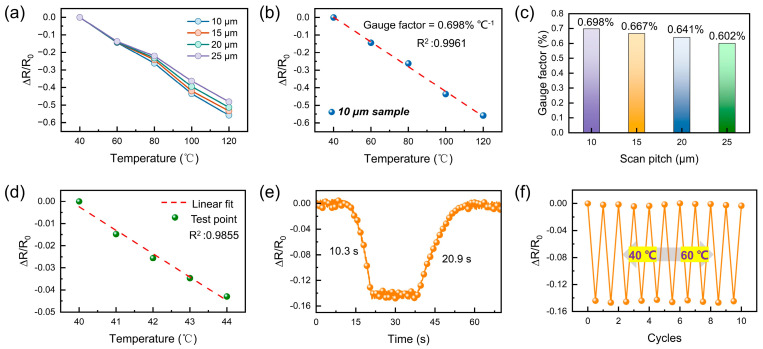
Evaluation of the sensing performance of the flexible temperature sensor based on FLIG. (**a**) Relative resistance changes curves of the sensor under different scanning spacings (10–25 μm) within the temperature range of 40–120 °C. (**b**) Electrical signal response and linear fitting results of the sensor within the temperature range of 40–120 °C. (**c**) Comparison of sensitivity coefficients of the sensors prepared with different scanning spacings. (**d**) Resolution ability of the sensor for weak temperature changes and linear fitting results. (**e**) Switching the response/recovery time test of the sensor from 40 °C to 60 °C. (**f**) Stability test of the sensor under temperature cycling ranging from 40 °C to 60 °C.

In order to verify the thermal response characteristics of the constructed flexible temperature sensors, the relative resistance change behavior (ΔR/R_0_) of the devices at different scanning spacings was systematically measured over a temperature range of 40–120 °C. The temperature response curves of the devices are shown in Figure 4a. The temperature response curves for four sets of samples with scanning spacings of 10 μm, 15 μm, 20 μm, and 25 μm, respectively. All the devices under scrutiny manifest typical negative temperature coefficient (NTC) characteristics; that is to say, the resistance value undergoes a gradual decrease in conjunction with an increase in temperature. This phenomenon is primarily attributable to the thermal excitation of the FLIG material during the temperature rise process. This process has been shown to enhance carrier mobility and interface conduction effect, thereby reducing the overall resistance. A thoroughgoing comparison reveals that the samples with 10 μm and 15 μm spacing exhibit larger response amplitude and better linearity. This finding suggests that the dense and uniform FLIG structure is more conducive to the construction of a continuous and stable conductive network, which in turn improves the response sensitivity to thermal excitation. This outcome is consistent with the aforementioned SEM morphology analysis, which further verifies the synergistic effect of smaller scanning spacing in structure optimization and performance enhancement. Furthermore, the linear fitting of the temperature response curve (Figure 4b) demonstrates that the 10 μm sample demonstrates a gauge factor of up to 0.698% °C^−1^ with a goodness-of-fit R^2^ of 0.9961, indicating that it exhibits excellent and predictable thermal response properties. The sensitivity coefficients at varying scanning spacings are compared in Figure 4c, and the results demonstrate that the sensitivity significantly increases with decreasing scanning spacings, thereby further confirming the critical role of the uniformity and connectivity of graphene networks in determining thermal response performance at reduced spacings.

As demonstrated in Figure 4d, the developed flexible temperature sensor demonstrates the capability to discern temperature fluctuations as low as 1 °C, attaining an electrical response linearity R^2^ of 0.9855 from 40 °C to 44 °C. In order to further evaluate the sensor’s ability to respond to a dynamic temperature stimulus, Figure 4e shows the real-time response curve of the device as the temperature rises from 40 °C to 60 °C and then recovers to the initial state. The findings demonstrate that the sensor demonstrates an adequate response rate and recovery capability in response to temperature fluctuations, with a response time of 10.3 s and a recovery time of 20.9 s, both of which are within the acceptable range for wearable thermal sensing applications. Both of these materials are within the acceptable range for wearable thermal sensing applications. This can be attributed to the fast thermal diffusion and enhanced interface sensitivity that is brought about by the porous structure of FLIG. The sensor is thus able to capture temperature changes and output stable signals on time. Furthermore, to evaluate the signal consistency and long-term stability of the device in real-world application scenarios, multiple rounds of temperature cycling were performed. The relative rate of change in resistance of the temperature sensor was measured by placing it on a heated table at a cyclically varying temperature between 40 °C and 60 °C. After the temperature had stabilized, the results are shown in Figure 4f. These results demonstrate the temperature sensor’s exceptional cyclic stability and response repeatability, with no ΔR/R_0_ response degradation and minimal curve fluctuations observed after 10 complete temperature cycles. This performance is essential for the functionality of the flexible, wearable sensing system and demonstrates the effectiveness of the laser direct writing process used to fabricate the flexible structures in this study. This performance is pivotal for the functionality of flexible wearable sensing systems, underscoring the dependability of the laser direct-write process for the fabrication of flexible structures in this study.

In order to verify the applicability and functional scalability of the device in practical application scenarios, further thermal sensing demonstration experiments were carried out. These experiments were based on a flexible manipulator platform and simulated the contact and non-contact heat source stimulation conditions, respectively. The experiment demonstrates two things. Firstly, it demonstrates the responsiveness of FLIG flexible temperature sensors in integrated systems. Secondly, it provides intuitive verification of their application in terminal sensing modules. Examples of such modules include electronic skin and intelligent robots.

**Figure 5:**
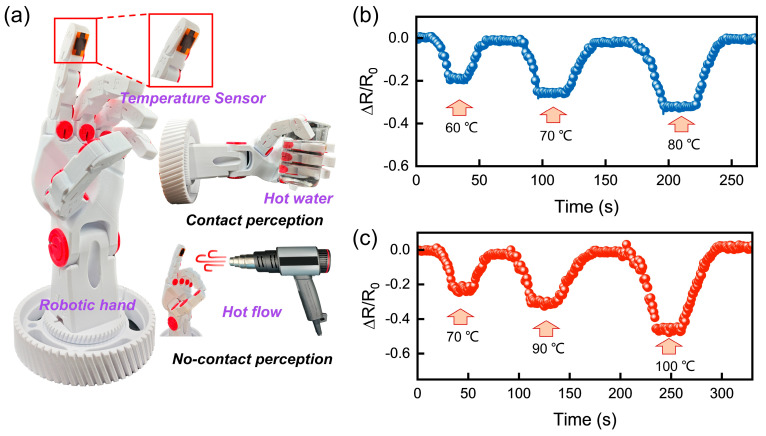
FLIG flexible temperature sensors integrated into a robotic hand system for thermal contact and non-contact sensing functions. (**a**) Schematic of the FLIG flexible temperature sensor integrated into the robotic hand. (**b**) contact sensing mode where the sensor responds to 60 °C, 70 °C, and 80 °C hot water stimuli. (**c**) non-contact sensing mode, where the sensor senses changes in a remote heat source.

As demonstrated in Figure 5a, the sensors were integrated onto the fingertip surface of the robotic hand and connected to the external data acquisition module via flexible wires. In the contact sensing test, the sensor was integrated into the robotic finger’s abdomen, and the acquisition of thermo-tactile signals was realized by directly grasping hot water glasses of varying temperatures (Figure 5b). As the temperature of the heat source increased from 60 °C to 80 °C, the resistance changes in the sensor output (ΔR/R_0_) remained stable and increased in a stepwise manner, indicating that it was able to accurately differentiate between the intensity of different contact heat sources. This stable and graded response characteristic indicates that the device not only possesses good temperature sensitivity but is also suitable for practical thermal contact monitoring scenarios with certain quantitative discrimination capability. Furthermore, non-contact thermal sensing experiments simulated heat flow field perturbations by blowing air into the surface of a mechanical finger with a hot air gun, with a 10 cm distance between the sensor and the heat source (Figure 5c). Despite the absence of direct contact with the heat source, the sensor demonstrated a swift and consistent response to temperature fluctuations, exhibiting a high degree of reproducibility in its output signals. This response was observed under conditions of hot air stimulation ranging from 70 °C to 100 °C. This ability to respond to heat flow perturbations over long distances is of particular importance for the development of smart terminals with “forward sensing” capabilities.

**Figure 6:**
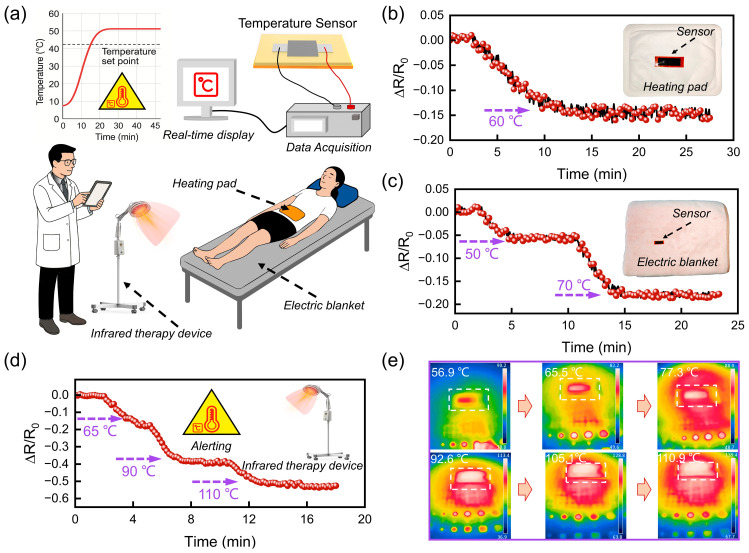
Demonstration of FLIG flexible temperature sensors for real-time health monitoring and high-temperature warning. (**a**) Schematic illustration of the sensor applied for patient temperature monitoring and predictive warning. (**b**) Real-time resistance response of the sensor during temperature variation induced by an electric heating pad. (**c**) Real-time resistance response of the sensor during heating by an electric blanket. (**d**) Real-time resistance response of the sensor under infrared physiotherapy stimulation. (**e**) Infrared thermographic image of the FLIG sensor under the irradiation from an infrared physiotherapist.

In order to provide further validation of the utility of FLIG flexible temperature sensors in medical and healthcare scenarios, a set of application demonstrations was designed in typical thermal therapy environments. These environments included electric heating pads, electric blankets, and infrared physical therapy devices. These devices are widely used in clinical rehabilitation, chronic pain management, and postoperative repair, where they enhance local blood circulation and tissue repair through mild heating. However, temperatures over 45 °C or prolonged exposure to low-temperature heat sources (38–45 °C) can result in scalding or low-temperature burns, which pose a heightened risk for elderly, unconscious patients. Consequently, it is imperative to acknowledge the significance of real-time temperature monitoring and the implementation of intelligent warning systems during thermal therapy. As demonstrated in Figure 6a, the FLIG sensor’s thin structure and bendable characteristics enable seamless integration into wearable platforms, such as electric heating pads, electric blankets, and rehabilitation suits. During operation, the resistance change induced by thermal stimulation is continuously converted into an electrical signal and transmitted to a computer via a data acquisition interface, thereby enabling real-time temperature monitoring and risk warning.

Figure 6b–d further illustrates the sensor’s response behavior under three types of typical thermal stimulation conditions. The heating process of the electric heating pad (Figure 6b) involves a gradual increase in resistance signal of the sensor, indicative of the device’s ability to precisely sense the slow warming process. The sensor’s resistance signal ultimately reaches a stable plateau at approximately 60 °C, exhibiting minimal fluctuations throughout the entire curve. This finding suggests that the device is well-suited for temperature tracking in low-speed thermal therapy scenarios. As illustrated in Figure 6c, the response characteristics of the electric blanket under graded heating conditions are demonstrated. The temperature increases from 50 °C to 70 °C. Consequently, the sensor signal displays a discernible step-like rise. Each temperature step corresponds to a stable output platform, exhibiting a brief response time and commendable repeatability. This phenomenon signifies its capacity to discern and adapt to dynamic temperature fluctuations.

In the more extreme infrared physical therapy test (Figure 6d), the temperature rapidly rises to 110 °C. However, the sensor maintains a linear, continuous resistance output trend without saturation or signal drift, thereby verifying the device’s operational stability under high-temperature thermal fields. As illustrated in Figure 6e, the infrared thermal image within the designated temperature range exhibits an incremental rise from 56.9 °C to 110.9 °C. The thermal distribution within the FLIG region remains consistent and uniform, devoid of any discernible accumulations of heat or localized anomalies. This observation serves to further substantiate the optimal thermal diffusivity and structural response consistency of the FLIG region. The findings indicate that the FLIG flexible temperature sensor demonstrates stable and repeatable response capability, as well as exceptional high-temperature adaptability under diverse thermal stimulus environments. This combination of attributes, along with its flexible, lightweight, and integrable characteristics, substantiates its extensive application prospects in smart rehabilitation devices, wearable health, monitoring systems, and high-temperature warning platforms.

## 4. Conclusions

In this study, a high-performance flexible temperature sensor was constructed on a PI flexible substrate based on FLDW technology, realizing the strategy of preparing FLIGs without a mask, at low cost, and digitally. The effect of laser scanning pitch on the structural morphology and sensing performance of FLIG was systematically investigated. The results show that a smaller pitch (10–15 μm) can obtain a denser porous graphene structure with better connectivity, which significantly improves the temperature response performance of the device. Under the optimal parameter conditions, the fabricated sensors exhibit good thermal response characteristics in the range of 40–120 °C, with temperature sensitivities up to 0.698%^−1^. Beyond the system performance evaluation, the sensor was successfully integrated into a flexible manipulator system, demonstrating both contact and non-contact thermal sensing. FLIG-based flexible temperature sensors provide an effective path for the construction of flexible thermal sensors with high sensitivity, high stability, and integrated application capabilities, and lay a technical foundation for their engineering and promotion in the fields of smart sensing, electronic skin, and wearable devices.

## Data Availability

The original contributions presented in the study are included in the article, further inquiries can be directed to the corresponding author.
